# The impact of quality control on cortical morphometry comparisons in autism

**DOI:** 10.1162/imag_a_00022

**Published:** 2023-10-06

**Authors:** Saashi A. Bedford, Alfredo Ortiz-Rosa, Jenna M. Schabdach, Manuela Costantino, Stephanie Tullo, Tom Piercy, Lifespan Brain Chart Consortium, Meng-Chuan Lai, Michael V. Lombardo, Adriana Di Martino, Gabriel A. Devenyi, M. Mallar Chakravarty, Aaron F. Alexander-Bloch, Jakob Seidlitz, Simon Baron-Cohen, Richard A.I. Bethlehem

**Affiliations:** Autism Research Centre, Department of Psychiatry, University of Cambridge, Cambridge, United Kingdom; Lifespan Brain Institute, The Children’s Hospital of Philadelphia and Penn Medicine, Philadelphia, PA, United States; Department of Child and Adolescent Psychiatry and Behavioral Science, The Children’s Hospital of Philadelphia, Philadelphia, PA, United States; Cerebral Imaging Centre, Douglas Mental Health University Institute, Montreal, Canada; Integrated Program in Neuroscience, McGill University, Montreal, Canada; Department of Psychiatry, University of Cambridge, Cambridge, United Kingdom; The Margaret and Wallace McCain Centre for Child, Youth & Family Mental Health and Azrieli Adult Neurodevelopmental Centre, Campbell Family Mental Health Research Institute, Centre for Addiction and Mental Health, Toronto, Canada; Department of Psychiatry and Autism Research Unit, The Hospital for Sick Children, Toronto, Canada; Department of Psychiatry, Temerty Faculty of Medicine, University of Toronto, Toronto, Canada; Department of Psychiatry, National Taiwan University Hospital and College of Medicine, Taipei, Taiwan; Laboratory for Autism and Neurodevelopmental Disorders, Center for Neuroscience and Cognitive Systems, Istituto Italiano di Tecnologia, Rovereto, Italy; Autism Center, Child Mind Institute, New York City, NY, United States; Department of Psychiatry, McGill University, Montreal, Canada; Department of Biomedical Engineering, McGill University, Montreal, Canada; Department of Psychiatry, University of Pennsylvania, Philadelphia, PA, United States; Cambridge Lifetime Asperger Syndrome Service (CLASS), Cambridgeshire and Peterborough, United Kingdom; Brain Mapping Unit, Department of Psychiatry, University of Cambridge, Cambridge, United Kingdom; Department of Psychology, University of Cambridge, Cambridge, United Kingdom

**Keywords:** structural MRI, quality control, FreeSurfer, cortical thickness, autism

## Abstract

Structural magnetic resonance imaging (MRI) quality is known to impact and bias neuroanatomical estimates and downstream analysis, including case-control comparisons, and a growing body of work has demonstrated the importance of careful quality control (QC) and evaluated the impact of image and image-processing quality. However, the growing size of typical neuroimaging datasets presents an additional challenge to QC, which is typically extremely time and labour intensive. One of the most important aspects of MRI quality is the accuracy of processed outputs, which have been shown to impact estimated neurodevelopmental trajectories. Here, we evaluate whether the quality of surface reconstructions by FreeSurfer (one of the most widely used MRI processing pipelines) interacts with clinical and demographic factors. We present a tool, FSQC, that enables quick and efficient yet thorough assessment of outputs of the FreeSurfer processing pipeline. We validate our method against other existing QC metrics, including the automated FreeSurfer Euler number, two other manual ratings of raw image quality, and two popular automated QC methods. We show strikingly similar spatial patterns in the relationship between each QC measure and cortical thickness; relationships for cortical volume and surface area are largely consistent across metrics, though with some notable differences. We next demonstrate that thresholding by QC score attenuates but does not eliminate the impact of quality on cortical estimates. Finally, we explore different ways of controlling for quality when examining differences between autistic individuals and neurotypical controls in the Autism Brain Imaging Data Exchange (ABIDE) dataset, demonstrating that inadequate control for quality can alter results of case-control comparisons.

## 1 Introduction

It is well established that magnetic resonance imaging (MRI) quality affects neuroimaging-derived neuroanatomical measures (Gilmore et al., 2021). MRI quality comprises multiple components, including head motion, imaging artefacts, and image processing outputs. Quality of the original image is often the starting point for quality control analyses as it affects all subsequent downstream analysis. Of particular concern when it comes to this raw image quality is in-scanner head motion, which has been consistently shown to affect estimates of brain structure (Alexander-Bloch et al., 2016; Makowski et al., 2019; Pardoe et al., 2016; Reuter et al., 2015; Savalia et al., 2017; Tisdall et al., 2016) as well as function (Goto et al., 2016; Power et al., 2012; Satterthwaite et al., 2012; van Dijk et al., 2012) and connectivity (Bastiani et al., 2019; Baum et al., 2018). For example, estimates of cortical thickness, surface area, and volume have consistent, regionally dependent relationships with motion (Alexander-Bloch et al., 2016; Pardoe et al., 2016; Reuter et al., 2015; Savalia et al., 2017). In addition to motion, other factors such as scanning artefacts, intensity inhomogeneities, and geometric and susceptibility-related distortions also impact image quality (Savalia et al., 2017). However, errors in image processing outputs and surface reconstructions further downstream also significantly impact and distort estimates of neuroanatomy, and in particular neurodevelopmental trajectories (Ducharme et al., 2016; Rosen et al., 2018; Savalia et al., 2017). Raw and processed output quality are, to an extent, interdependent, as accurate image segmentation and surface reconstruction relies on good raw image quality. However, image processing can fail or produce errors even in excellent quality images; thus, it is important to consider both aspects. Critically, image quality of all kinds, and head motion in particular, are highly correlated with demographic characteristics such as age, sex, as well as variables of interest such as diagnostic status in clinical cohorts (Alexander-Bloch et al., 2016; Pardoe et al., 2016; Savalia et al., 2017), and there is evidence that these biases also permeate case-control comparisons (Bedford et al., 2020; Yendiki et al., 2014). Although these issues are becoming more widely acknowledged, there is currently no “gold standard” of quality control (QC) methods, especially when it comes to evaluating image processing outputs. Detailed QC procedures are also rarely reported, making quantitative evaluations across studies difficult.

Few extensive and detailed manual quality control protocols have been explicitly published (Backhausen et al., 2016). While authors summarise QC procedures in Methods Sections or Supplementary Results (Bedford et al., 2020; Pardoe et al., 2016), often little detail is given. Some papers have provided and assessed detailed protocols for QC of image processing outputs, often of FreeSurfer (Fischl, 2012), one of the most popular and widely used tools for cortical surface reconstruction. For example, Visual QC (Raamana et al., 2021) and a QC protocol provided by the ENIGMA consortium (*Protocol for Quality Control and Summary Statistics « ENIGMA*, n.d.) provide detailed guidelines and a framework in which to view and rate images and their FreeSurfer outputs. While these protocols offer a comprehensive and useful tool for evaluating scans and surface reconstructions, they are time consuming, and hence may be impractical for very large datasets. This highlights the need for rigorous yet efficient manual QC methods for outputs of FreeSurfer and similar processed images and tools.

The increasing sample sizes typically used in neuroimaging studies (Bethlehem et al., 2022; Di Martino et al., 2017; Marek et al., 2022; Postema et al., 2019; Thompson et al., 2014; Weiner et al., 2015; Van Essen & Glasser, 2016) is another barrier to implementing thorough and rigorous QC. Manual QC is both time and labour intensive, and it requires expert raters and/or extensive training of individuals to examine and assess both raw scans and post-processed outputs, as well as assessment of inter-rater reliability (Ai et al., 2021; Bedford et al., 2020; Rosen et al., 2018). With samples routinely in the thousands or even tens of thousands, this may be impractical or infeasible. In recent years, various alternative, automated QC methods have been proposed. For example, FreeSurfer’s Euler number, a measure representing the topological complexity of the cortical surface reconstruction, is regarded as a good proxy for image quality, correlating highly with manual quality ratings, as well as regional measures of cortical thickness (Rosen et al., 2018). Other automated methods of quality assessment have combined multiple automatically derived quality metrics such as detection of artefacts, background intensity distribution, and signal-to-noise ratio (Mortamet et al., 2009; Shehzad et al., 2015). Building on these tools, the now widely used MRIQC (Esteban et al., 2017) provides comprehensive automated reports of image quality, and prediction of manual quality ratings, based on various (raw) image quality metrics, which also include measures of noise, entropy (indicative of motion), statistical properties, cortical features and extreme values, and specific artefacts. Another recently developed tool, Qoala-T (Klapwijk et al., 2019), focuses on post-processing quality, providing an automated binary include/exclude label to FreeSurfer outputs, along with a probability score indicating the estimated scan quality. Another approach is to use “citizen science,” combined with manual expert ratings and machine learning, to generate thousands of QC ratings, lessening the burden on researchers. This approach has resulted in the Swipes for Science initiative (swipesforscience.org), which crowd-sources QC ratings (binary pass/fail classification) of raw images, and also accounts for variations in quality of ratings by different raters (Keshavan et al., 2019). However, these raters are rarely experts, and receive minimal to no training on the ratings and criteria. The trade-off between efficiency and rigour when it comes to comparing automated to manual QC procedures is also still an open question which requires further investigation.

The lack of consensus and standardised methods is particularly problematic for large publicly available datasets, as it makes comparisons between different studies using the same datasets challenging and it is unclear to what extent inconsistencies in results are due to inconsistent QC methods or standards. This is a particularly salient issue in neurodevelopmental imaging, as inadequate image quality has been shown to impact findings (Ducharme et al., 2016; Savalia et al., 2017), and participants with neurodevelopmental conditions such as autism are more susceptible to image quality issues (often due to motion) than neurotypical individuals (Alexander-Bloch et al., 2016; Bedford et al., 2020; Pardoe et al., 2016). Without adequate QC, there is a high risk of spurious correlations or group differences, as well as true effects being obscured by motion or quality issues. Numerous studies have used the Autism Brain Imaging Data Exchange (ABIDE) (Di Martino et al., 2014, 2017) to examine case-control differences related to autism, using both structural and functional measures (Bedford et al., 2020; Bethlehem et al., 2017, 2020; Floris et al., 2018; Haar et al., 2016; Khundrakpam et al., 2017; Kohli et al., 2019; Nielsen et al., 2014; Olafson et al., 2021; Ray et al., 2014; Schaer et al., 2015; Turner et al., 2016; Valk et al., 2015). Although there is some convergence of these findings, there are also conflicting and inconsistent findings between studies, which may in part be due to differences in QC procedures and thus differences in the final sample. The ABIDE Preprocessed repository (http://preprocessed-connectomes-project.org/abide/) includes quality ratings for ABIDE I based on the Quality Assessment Protocol (QAP) by Shehzad et al. (2015), though these do not provide a logical cut-off point or threshold. The issue of how extensively variations in quality impact findings related to neurodevelopmental and psychiatric conditions urgently warrants further investigation.

Given the need for systematic, rigorous, and reproducible QC methods, we aimed to develop a quick and efficient yet thorough tool for QC of FreeSurfer surface reconstructions. Our FSQC tool allows for multiple ratings per participant that take only a few seconds, and also captures aspects of raw image quality, specifically motion, which is included in the overall rating of a participant. Thus, this tool can be used either as a stand-alone method that assesses some of the most important aspects of quality, or as a complementary method to other existing, perhaps automated, QC tools. We then aimed to validate our FSQC metric against other QC methods in the ABIDE dataset, both manual and automated, to attempt to quantify the trade-off and comparability between methods. Finally, we assessed the impact of QC on regional estimates of cortical morphometry, and examined the interaction between quality and diagnostic status in the context of autism. Importantly, we demonstrate that failing to account for quality can have subtle but significant impacts on apparent case-control differences, and thus has the potential to be an important confound in studies of neurodevelopmental or psychiatric conditions.

## 2 Methods

### 2.1 Sample

The ABIDE dataset consists of neuroimaging, demographic, and clinical data from 2226 individuals (1060 autistic individuals and 1166 neurotypical controls), aged 5-64 years (1804 assigned-males-at-birth, 422 assigned-females-at-birth). The ABIDE repository includes two waves of data aggregation (ABIDE I and II), from a total of 24 international sites. Participant demographics and acquisition information have been previously described in detail (Di Martino et al., 2014, 2017).

### 2.2 FreeSurfer QC method and generation of images

#### 2.2.1 Processing with FreeSurfer

All T1-weighted structural scans were processed with FreeSurfer 6.0.1 (see Di Martino et al. (2014, 2017) for details on ABIDE acquisition). A subset of 50 participants were also processed with FreeSurfer 7.1 for comparison with newer methods. Cortical parcellations were derived using the Glasser (Glasser et al., 2016) and Desikan-Killiany (Desikan et al., 2006) atlases. Glasser parcellations were derived for each participant by resampling the Glasser parcellation template to FreeSurfer fsaverage, and from there back to individual subject space, using FreeSurfer’s surface-based registration. Recent work has demonstrated that atlases with higher-dimensional cortical representation are able to capture a higher proportion of trait variance accounted for by the cortical measures (Fürtjes et al., 2023). Thus, we chose to present our main results using the Glasser parcellations, to provide a more fine-grained and detailed profile of spatial relationships. However, because the Glasser parcellations are multimodally derived, we also conducted all analyses using the Desikan-Killiany parcellations, a structurally derived atlas. In order to enable comparison with other studies that use the Desikan-Killiany parcellations, and to allow comparison of results between parcellation schemes, these results are also presented in the Supplementary Materials.

#### 2.2.2 Generation of FSQC images

**Fig. 1.**
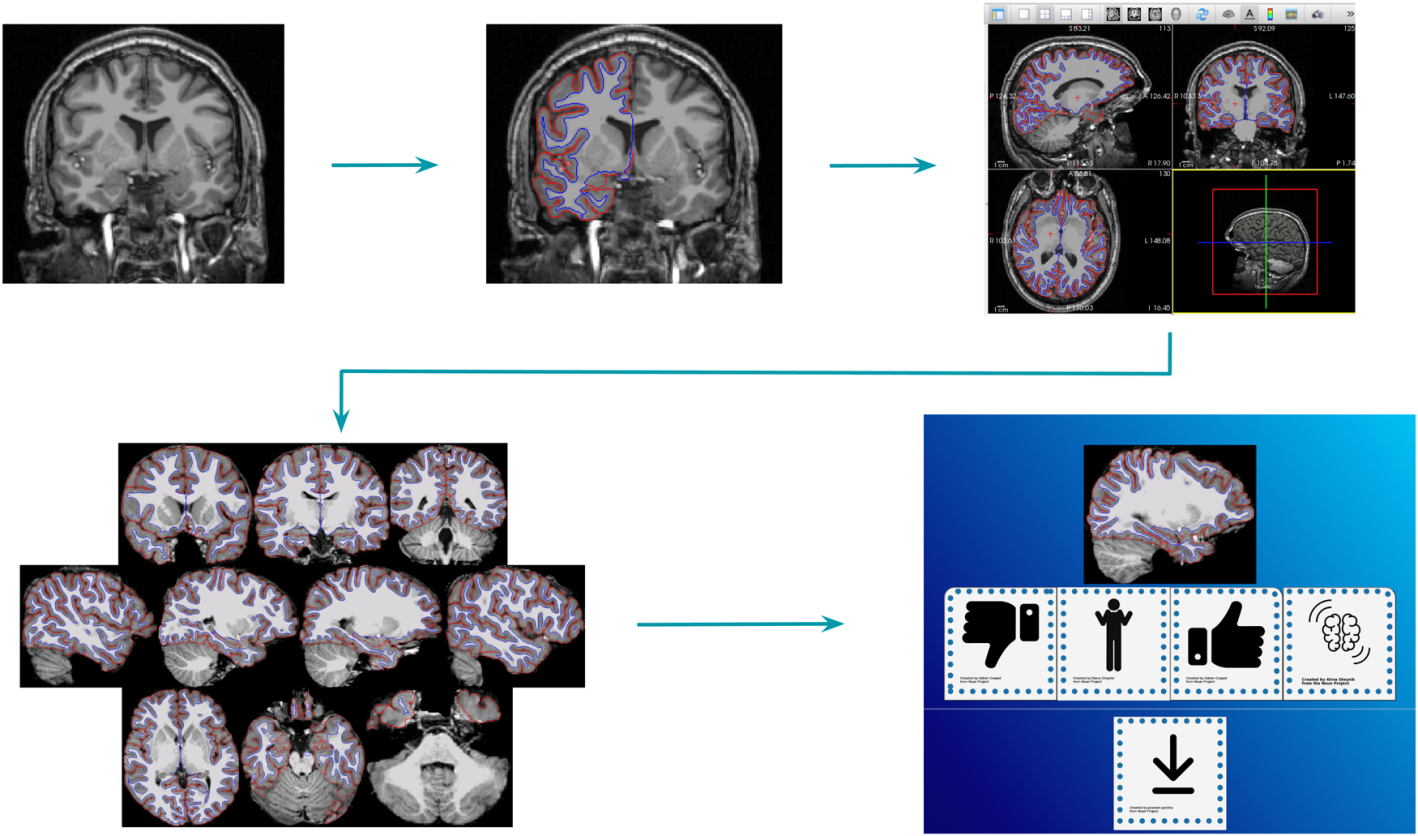
FSQC image generation workflow. From left to right: T1 images were processed with FreeSurfer 6.0.1 and displayed in FreeView with pial and white matter surfaces overlaid on the T1 image (both hemispheres). Screenshots were automated and taken at predefined, consistent coordinates, for a total of 10 images per participant. Images were then displayed and rated in the Image-Rating app, and scores were averaged across all 10 images for each participant.

QC images were generated by overlaying the FreeSurfer-derived cortical surface boundaries on the participant’s T1 scan in FreeSurfer’s FreeView visualisation tool, and using the FreeView Screenshot function to generate screen captures at 10 different views and slices of the brain. The 10 slices (3 axial; 3 coronal; 4 sagittal; see Fig. 1) were chosen by selecting views which give a good representation of the whole brain, based on manual inspection of a few images. Slices were taken at intervals of roughly 20, without being too near to the edges of the brain as this may result in some participants having blank images if their heads are in a slightly different position. Three slices each were selected for axial and coronal views, at roughly one quarter intervals across the brain, but four were selected for the sagittal view to avoid having images of the mid-section of the brain, and so that two views per hemisphere are captured. Based on the images that were manually reviewed slice by slice, this appears to give a good representation of quality of the raw image and reconstruction. This process was then automated in a virtual server window, with consistent coordinates specified for each participant for the 10 screenshots (code shared below). For comparison and to confirm that 10 slices is sufficient to get a good representation of the quality of the whole image, we also generated FSQC images for two participants of 20 slices instead of 10 (Supplementary Methods 1.1). We also note that the code for FSQC image generation is easily adaptable; thus, researchers can easily increase (or decrease) the number and position of slices if they wish to.

Prior to rating, each image was renamed using the MD5 message digest algorithm and images were randomly shuffled so ratings were not biased by other images from the same participant appearing in sequence. Participants were not divided by site, so as to also not be influenced by any particularities at a specific site. Images were then viewed in the Image-Rating QC application (https://github.com/sbedford0/FSQC/tree/main/imageratingQCApp), and assessed for accuracy of the cortical reconstruction (grey-white matter and grey matter-pial surface boundaries), as well as presence of motion in the raw T1 image on which the surfaces were overlaid. Each image (10 per participant) was rated individually on a scale of 1-4 (good - bad), corresponding to the following categories: good (1), minor error (2; i.e., often involving misestimation of boundaries restricted to one or two specific regions), visible motion (3; defined as ringing or rippling artefact visible at any point in the image, or blurring, resulting in unclear grey/white matter boundaries and reduced clarity of the image), and bad (4), indicating very poor surface reconstruction with multiple errors or large areas of missing cortex. “Bad” is rated as worse than “motion” to reflect the fact that some small amount of motion, confined to a small area of the image, may not render it unusable. See https://www.protocols.io/view/fsqc-protocol for images, detailed criteria, and examples of each score. Outputs from the Image-Rating app were recorded and downloaded in a csv file. Categorical ratings were then converted to the corresponding numerical rating (i.e., 1-4), and averaged across all 10 images for each participant, to give a final continuous score between 1 and 4 per participant. Thus, these scores provide a quality rating reflecting the accuracy of the FreeSurfer surface reconstructions as well as some indication of the presence of motion in the raw T1 image. We note that it is possible for a significant artefact or error to be visible in one slice only and thus still result in a very good score, though in our experience this is uncommon. We also maintain that the score would still be representative of the overall good quality of reconstruction; however, if a researcher would like to be more stringent, they are easily able to set their exclusion threshold accordingly, or to exclude participants based on individual image ratings.

### 2.3 Statistical analysis

#### 2.3.1 FSQC inter-rater reliability

Two raters (S.A.B. and R.A.I.B.) rated the entire dataset, and an average of the two scores was taken for each participant. To ensure reasonable inter-rater reliability, raters first rated a subset of 20 participants (200 images), which were compared. Scores were averaged across the 10 images for each participant, and for any participant that had a discrepancy greater than 1 between the two raters, the images were discussed and a consensus was agreed upon before moving on to the rest of the dataset. This consensus rating was also used to clarify any discrepancies or things that were not clear in the image rating protocol. To assess inter-rater reliability of the method and protocol across multiple raters, 4 additional raters, for a total of 6 raters (S.A.B., R.A.I.B., A.O.-R., J.S., A.F.A.-B., J.M.S.), assessed a subset of 50 participants (500 images); Spearman’s correlations and two-way ICC for agreement were calculated across all raters, on the average score for each participant.

The first main analysis (examining the effect of FSQC on cortical thickness, see below) compared both individual rater’s scores, as well as the average score across all raters to ensure consistency (Supplementary Methods 1.2). For all subsequent analyses using FSQC, the average scores between the two raters were used, to minimise the potential of bias by one specific rater and increase generalisability of our results. The same 50 participants used to assess inter-rater reliability were also processed using FreeSurfer 7.1. FSQC images were generated and rated by rater S.A.B., and FSQC ratings and Euler number were compared to the 6.0 outputs (Supplementary Methods 1.3).

#### 2.3.2 Timing of ratings

The FSQC tool provides a “deliberation time” in milliseconds for each image. In order to calculate an estimate of the time needed to score one participant, we calculated the median score per participant per rater. First, we removed any outliers at >5 median absolute deviations, to remove lengthy times due to the rater getting distracted or taking a break during ratings. Then, for each rater individually, we took the median score for each participant across the 10 images. Finally, we took the median score across participants for each rater, to give a representative range and average of rating times per participant.

#### 2.3.3 Relationship between different QC metrics

First, we sought to validate our FSQC method by examining the relationship between FSQC scores (averaged across 10 images per participant, and two raters) and other QC metrics. These included the FreeSurfer-derived Euler number (Rosen et al., 2018) (a measure of topological complexity; lower numbers indicate better quality); a manual score assessing the presence and amount of motion in each image (“Motion QC”; raters S.A.B., M.M.C., S.T. (Bedford et al., 2020), see https://github.com/CoBrALab/documentation/wiki/Motion-Quality-Control-%28QC%29-Manual); and another manual rating of overall image quality which was derived from and built upon “Motion QC” (“PondrAI QC”; raters M.C., G.A.D., see https://github.com/pondrai/PondrAIQC). We also included comparisons to two popular automated QC tools: MRIQC, an automated prediction of raw image quality, and Qoala-T, an automated classification of FreeSurfer output quality. Qoala-T was run according to the instructions at https://github.com/Qoala-T/QC. Qoala-T provides a binary classification of include or exclude, as well as a certainty score (0-100), with scores closer to 0 or 100 indicating higher certainty of the binary decision. We used the certainty score as a continuous measure to compare against FSQC and the other quality metrics. For MRIQC, we used the MRIQC Quality Metrics which are publicly released for the ABIDE II dataset and available for download on the ABIDE website at http://fcon_1000.projects.nitrc.org/indi/abide/abide_II.html#:~:text=ABIDE%20II%20MRI%20Data%20Quality%20Metrics. Since multiple MRIQCquality metrics are provided, and there is not one overall score intended to be used for thresholding, we assessed correlations with each metric, and present these in a correlation matrix. Finally, because MRIQC was only released with the ABIDE II repository, we also included a comparison to the Quality Assessment Protocol (Shehzad et al., 2015) metrics released with ABIDE Preprocessed (http://preprocessed-connectomes-project.org/abide/) for ABIDE I. These analyses and results are presented in the Supplementary Materials (Supplementary Methods 1.4).

Spearman correlations were run to assess the relationship between FSQC and each other metric.

#### 2.3.4 Demographic correlations

Since demographic factors are related to image quality (Alexander-Bloch et al., 2016; Bedford et al., 2020; Pardoe et al., 2016), we next investigated these relationships in our dataset. Of particular interest were age, sex-assigned-at-birth (hereafter “sex”), and diagnosis, as these variables are especially relevant to neuroimaging studies of autism and have been shown by previous work to correlate with image quality, and motion specifically (Alexander-Bloch et al., 2016; Pardoe et al., 2016; Reuter et al., 2015). To account for site differences, linear mixed-effects models were used to examine the impact of age, a quadratic term for age (age^2^), sex and diagnosis (with site as a random effect) on all quality metrics separately (FSQC, Euler, Motion QC, PondrAI QC, Qoala-T).

#### 2.3.5 Impact of QC on cortical morphometry

To examine and quantify the impact of image quality, as measured by all QC metrics, on different neuroanatomical measurements, we assessed the relationship between each QC measure and global neuroanatomical measure, including total cortical and subcortical grey matter volumes (cGMV and sGMV), total brain volume (TBV), total white matter volume (WMV), total ventricular volume, and mean cortical thickness. Linear mixed-effects models were used for all analyses, with site as a random factor, to account for inter-site variability and differences in the ABIDE dataset.

As previous work has demonstrated spatially dependent relationships with quality (Alexander-Bloch et al., 2016; Pardoe et al., 2016; Reuter et al., 2015), we next examined regional effects on cortical thickness (CT), surface area (SA), and cortical volume (CV). Relationships with subcortical phenotypes were not assessed as the surface reconstructions being rated in the FSQC tool include only the cortical surface boundaries. Analyses were initially run on all participants (i.e., no exclusions), to examine the relationship between different types of quality and cortical morphometry across the whole spectrum of quality. For these analyses, linear mixed-effects models were run for each parcellation across the brain, separately for CT, SA, and CV. All regression models included QC metric, age, age^2^, and sex as fixed effects, and site as a random effect, with CT/SA/CV as the dependent variable, for each region. Partial correlations were calculated to quantify the strength of the association between QC metric and neuroanatomical measure (e.g., FSQC and CT; motion QC and CV, etc). Because Qoala-T was the only metric in which higher values denote better (rather than poorer) quality, we multiplied each partial correlation for Qoala-T by -1 so that the direction of the relationship matched the other QC measures, and relationships with cortical measures could easily be compared across metrics. Results were corrected for multiple comparisons using the false discovery rate (FDR) across parcellations in all analyses. For subsequent analyses, we focus on FSQC, our newly developed quality metric, and Euler, a commonly used automated method.

Main analyses were run using Glasser parcellations to provide a more fine-grained comparison, with Supplementary Analyses also run using Desikan-Killiany parcellations, for comparison with previous work (Supplementary Methods 2.1). To ensure we were adequately accounting for site effects, the main analyses were repeated using a random-effects meta-analysis for comparison, and to assess heterogeneity of results across sites (Supplementary Methods 2.2). We also attempted to replicate these analyses in multiple datasets. These included a larger, more representative dataset of 74,647 individuals (that has been previously used (Bethlehem et al., 2022)), and multiple publicly available neurodevelopmental datasets (the Child Mind Institute’s (CMI) Healthy Brain Network, the ADHD200 dataset, and the Province of Ontario Neurodevelopmental (POND) Network; Supplementary Methods 2.3). Finally, we conducted a variance partitioning analysis (Hoffman & Schadt, 2016) to evaluate the relative contribution of image quality to the total variance explained, compared to factors such as diagnosis, age, sex, and site (Supplementary Methods 2.4).

#### 2.3.6 Exclusion/thresholding analyses

Quality control scores are often used as a way to exclude data of poor quality; for example, previous studies using the Euler number as a QC metric recommend a study-specific threshold (Rosen et al., 2018). To evaluate the impact of different quality thresholds on the relationship with cortical morphology and existence of group-level differences, and to assess the extent to which results were driven by participants with the worst or more extreme image quality, we conducted a thresholding analysis, examining the impact of quality (FSQC and Euler number) at cut-offs of varying stringency. All analyses were conducted using linear mixed-effects models, with site as a random factor.

For FSQC, we chose score thresholds in increments of 0.5 points (3, 2.5, 2, 1.5). The same models and analyses described above were re-run after excluding participants at each of these thresholds, for each cortical phenotype. For Euler number, there were less obvious cut-off points than for FSQC, and there is no universally accepted threshold of good versus poor quality data. Therefore, we used median absolute deviations (MAD) to determine various thresholds for these analyses. The range of Euler number in our dataset was 7-775 (mean = 129.7; median = 103.0; standard deviation = 99.4). The relationship between Euler and each cortical phenotype was assessed after thresholding at 1, 2, and 3 MADs, and half points in between (corresponding to Euler numbers of 139, 174, 210, 245, 281, and 317). Due to the significant differences and variability between sites, Supplementary Analyses were also conducted applying MAD-based cut-off points calculated and applied individually per site, rather than across the whole sample (Supplementary Methods 3.1).

Additional sensitivity analyses were performed, including comparing high and low quality based on a median FSQC split, and thresholding based on the top percentage of scores (applied to the whole sample and per site) (Supplementary Methods 3.2-3.3).

#### 2.3.7 Interaction between image quality and diagnosis

As image quality differs by diagnostic status and impacts neuroanatomical estimates (Alexander-Bloch et al., 2016; Pardoe et al., 2016; Reuter et al., 2015), it is likely that inadequate accounting for quality will lead to inaccurate conclusions relating to diagnostic differences. To this end, we examined differences in cortical morphometry between autistic individuals and controls with different methods of accounting for quality, and at different quality thresholds. First, we examined group differences in CT, SA, and CV without accounting for quality, using linear mixed-effects models with diagnosis, age, age^2^, and sex in the model, and site as a random factor. Next, the same models were run with the addition of FSQC or Euler number as a covariate to assess the impact of controlling for quality, as well as thresholding by both FSQC (at 2.5) and Euler (at 2 MAD).

Supplementary Analyses for CT replicated these results in the CMI and POND datasets (Supplementary Methods S4.1). Further Supplementary Analyses examined diagnostic effects after thresholding by FSQC or Euler at various cut-off points (FSQC: 3, 2.5, 2, and 1.5; Euler: 1, 2, and 3 MADs; Supplementary Methods 4.2), as well as the effect of diagnosis on CT after thresholding by FSQC and also controlling for Euler (Supplementary Methods 4.3). Finally, we examined the interaction between diagnosis and FSQC or Euler on CT (Supplementary Methods 4.4).

## 3 Results

### 3.1 Inter-rater reliability

**Fig. 2.**
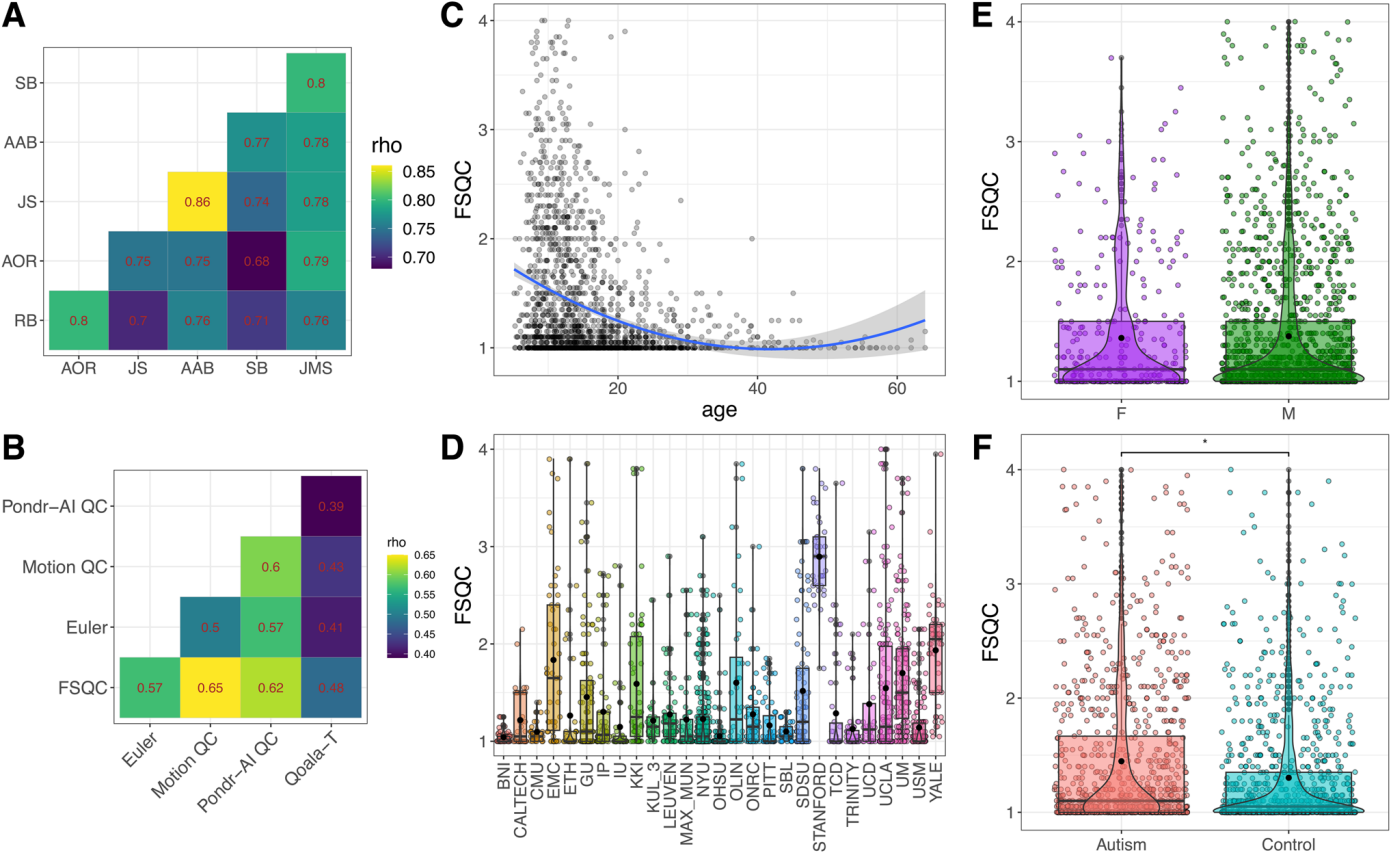
(A) Inter-rater correlation matrix for FSQC ratings for a subset of 50 participants (500 images). All pairs of raters were significantly correlated with each other between 0.7-0.8 rho. (B) Correlations between different QC metrics. Because Qoala-T is reverse coded relative to the other metrics, the absolute values are shown for the Qoala-T correlations. All measures were significantly correlated with each other. (C) Relationship between FSQC and age. A significant effect of age was observed in which younger participants had lower quality ratings. (D) FSQC score distributions by site. There was significant variability in quality across sites. (E) Box and violin plot of FSQC distributions for males and females. There was no significant sex difference in FSQC. (F) Box and violin plot of FSQC distributions by diagnosis. Autistic participants had significantly higher FSQC scores (i.e., lower image quality) relative to controls (p < 0.0001, *d* = -0.27). Box plots represent the interquartile range, the middle line denotes the median, and the black dot represents the mean. The curves of the violin plots show the distribution and density estimate of FSQC scores for each group.

For the subset of 50 participants, the ICC was moderate, at 0.68 for all 6 raters, including the two more experienced raters. Spearman correlations calculated between each pair of raters ranged from 0.68-0.86 (see Fig. 2A). For the whole dataset, the inter-rater Spearman correlation was 0.63 between raters S.A.B. and R.A.I.B.

Results of the impact of FSQC on CT were nearly identical when using each rater’s scores separately (S.A.B. and R.A.I.B.), and the average of the two scores (see Supplementary Figure S1.2). FSQC ratings and Euler number were largely consistent and highly correlated with FS7.1 outputs (Supplementary Results S1.3).

### 3.2 Timing of ratings

The median time to rate one participant (10 images) was 20.4 seconds across all 6 raters (range: 5.0-53.7 seconds) and 7.1 seconds across our two main, trained raters (range 5.0-9.3 seconds).

### 3.3 Relationship between different QC metrics

FSQC was significantly correlated with all other measures, with the exception of MRIQC’s EFC and Cortical contrast. Correlations with all metrics except MRIQC were moderate (Euler number (rho = 0.57, p < 0.0001); Motion QC (rho = 0.65, p < 0.0001); PondrAI QC (rho = 0.62, p < 0.0001); Qoala-T (-0.48, p < 0.0001)). (Note that the correlation between FSQC and Qoala-T is negative because higher values denote lower quality in FSQC but higher quality in Qoala-T.) Correlations with MRIQC quality metrics ranged from rho = -0.03-0.16. Pairwise correlations are shown in Figure 2B. The QAP IQMs showed similarly weak correlations with manual QC methods and Euler number (Supplementary Figure S1.4).

### 3.4 Demographic correlations

We assessed the relationship between each metric and demographic variables previously reported to be highly correlated with image quality (Fig. 2C-F). For all quality measures, autistic participants had significantly lower image quality relative to controls (all p < 0.01; Cohen’s *d* = -0.14 - -0.29). For all metrics, there was also a significant effect of age and age^2^ (p < 0.0001). However, when we examined the relationship between age and quality in young and old groups after performing a median split, both groups showed a negative relationship, reflecting lower image quality in younger participants, consistent with previous studies (Alexander-Bloch et al., 2016; Pardoe et al., 2016). For motion QC (p = 0.004, Cohen’s *d* = 0.16) and Qoala-T (p = 0.0001, Cohen’s *d* = -0.23) only, there was a significant effect of sex, where males had significantly lower quality scans than females. To assess whether this was due to differences in brain or head size, we repeated these analyses with estimated total intracranial volume (eTIV) in the model. This did not change the results in any model. However, eTIV had a significant effect on Euler number and Qoala-T (p < 0.001), but not any of the manual metrics. Image quality, across all metrics, also differed significantly by site (p < 0.0001).

### 3.5 Impact of QC on cortical morphometry

**Fig. 3.**
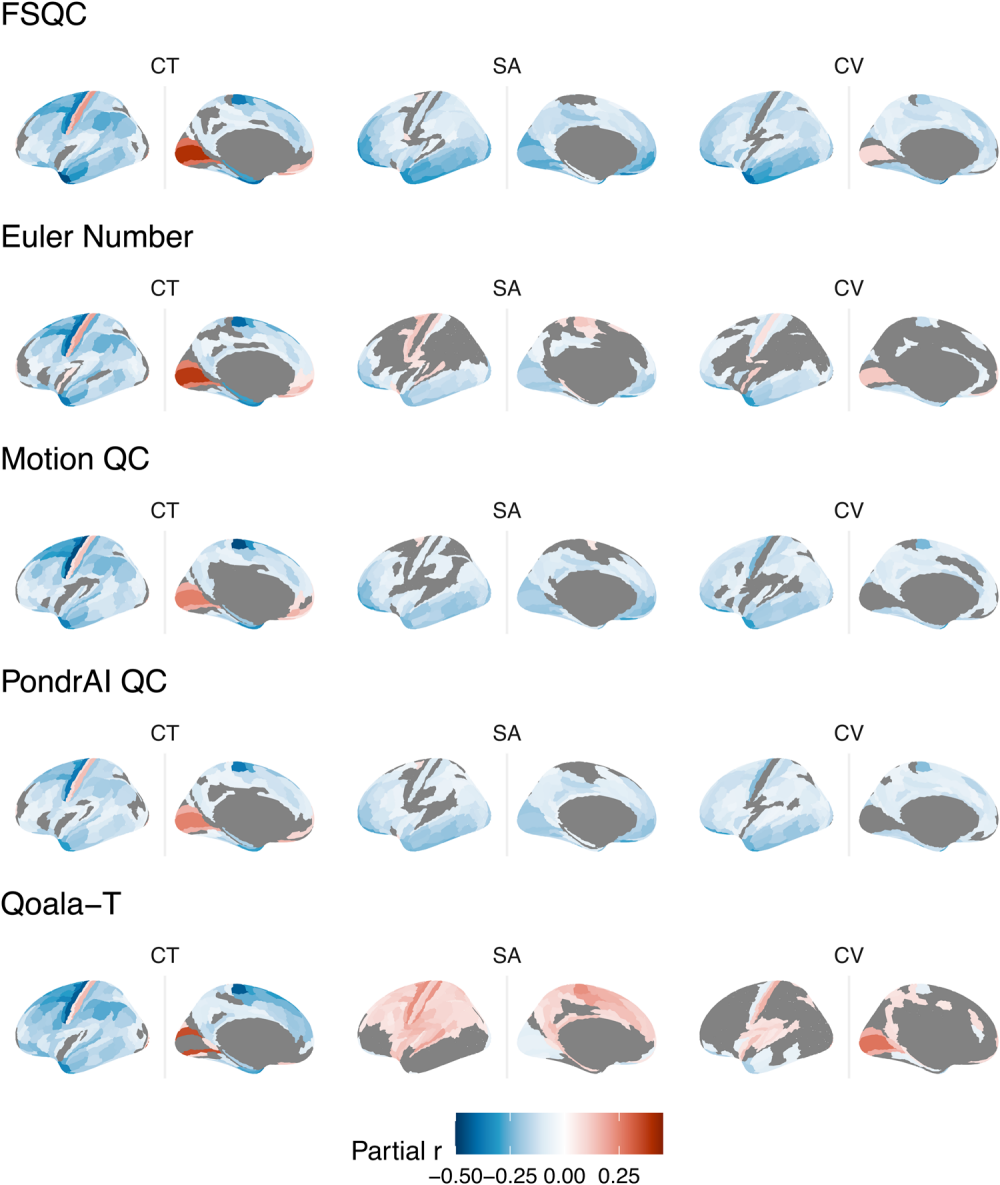
Associations between QC metrics and regional cortical morphometry. There was a significant relationship between image quality and neuroanatomical estimates across much of the cortex for all metrics and phenotypes. Relationships were largely negative and strongest for cortical thickness. Spatial patterning of results was highly similar across most metrics, with the exception of SA and CV for Qoala-T, which showed largely positive relationships, in contrast to the other metrics.

FSQC was significantly but weakly correlated with global brain measures of total cortical GMV, WMV, subcortical GMV, and TBV at a Bonferroni-corrected threshold of p < 0.008 for six comparisons (rho = -0.07 - -0.16), but not with mean CT or ventricular volume (see Supplementary Table S1 for all correlations). Regional analyses revealed significant associations across much of the cortex for all cortical phenotypes and QC metrics, passing 5% FDR (partial r = -0.49-0.43). Associations were largely negative, denoting apparent decreases in cortical measures with lower quality (higher scores), though strong positive relationships (increased measures with lower quality) were observed in some regions and analyses. Each phenotype showed distinct spatial relationships with quality; however, spatial patterning across the cortex was, for the most part, strikingly similar between metrics within each phenotype, in particular for cortical thickness. This was somewhat less true for SA and CV; spatial patterning was extremely similar across the three manual ratings, with slight differences observed for Euler number, but for Qoala-T, largely positive relationships were observed. Despite this, in the unthresholded maps, some spatial homology can still be observed across metrics, in particular with Euler. Results of regional analyses are shown in Figure 3, showing partial r values thresholded for significant regions (surviving 5% FDR). For maps of all (including non-significant) partial r values across the cortex, see Supplementary Figure S2.

Of the three cortical phenotypes, the strongest associations overall were observed for CT, and as such were the main focus of subsequent analyses (with CV and SA results reported in Supplementary Materials). Spatial maps for CT were strikingly similar across all five metrics. The strongest negative correlations between CT and image quality (across metrics) were observed in lateral superior frontal (including precentral gyrus), parietal, and inferior temporal regions, with widespread weaker, but still significant, negative correlations across much of the frontal, parietal, and temporal cortices. Significant positive correlations were observed in the medial occipital and ventromedial prefrontal cortices for all metrics, as well as in the postcentral gyrus (Fig. 3).

The strongest significant negative correlations for surface area were observed in inferior (medial and lateral) frontal and temporal cortices, as well as the medial occipital cortex. Correlations and spatial patterning were again mostly consistent across QC metric, with the exception of a larger number of positive correlations, and slightly fewer significant correlations overall, observed for Euler number, and to an even greater extent in Qoala-T. For Motion QC and PondrAI QC, almost no positive correlations reached significance, and in FSQC, only two or three disparate regions (including the postcentral gyrus) showed positive significant correlations. For Euler number, by contrast, significant positive correlations were observed in regions including the pre- and postcentral gyrus, medially and laterally, as well as the superior temporal gyrus, and for Qoala-T positive correlations were observed across much of the frontal and parietal lobes (Fig. 3).

Spatial patterning for cortical volume was again very similar across metrics, with slight differences in Euler, and with the exception of Qoala-T. The medial occipital cortex was significantly positively correlated with QC in FSQC, Euler, and Qoala-T, but did not reach significance in the other two metrics, though subthreshold correlations were also positive. For the three manual metrics, significant but weak negative correlations were observed across much of the cortex, and most strongly in inferior temporal and frontal regions, and the precentral gyrus. For Euler and Qoala-T, less regions met significance, including large areas of the frontal and parietal cortices. Most significant correlations were still negative for Euler, though positive correlations were observed in the postcentral gyrus, medial prefrontal cortex, and left hippocampus. For Qoala-T, significant positive correlations were also observed in the superior temporal cortex, postcentral gyrus, and medial parietal regions. Significant negative correlations with Qoala-T were observed in inferior temporal areas, in line with the other metrics (Fig. 3).

Desikan-Killiany parcellations yielded consistent results in almost all regions, with the exception of a few areas in which there was a change from positive to negative effect size in adjacent regions in the Glasser parcellations (e.g., postcentral gyrus, V1), which were obscured by the coarser parcellations (Supplementary Results 2.1). Results of the meta-analytic technique also displayed consistent spatial patterning. Our replication analyses also yielded largely consistent results across datasets (Supplementary Results S2.2-2.3). The variance partitioning analysis indicated FSQC and Euler contributed a relatively small portion of the variance, but larger than diagnosis (Supplementary Results S2.4).

Almost all analyses showed the strongest effects for cortical thickness, consistent with previous work suggesting that CT is more susceptible than other cortical estimates to impacts of image quality and motion (Pardoe et al., 2016). Consequently, and for clarity, subsequent analyses will focus primarily on the relationship between CT and image quality. For cortical surface area and volume results, see Supplementary Results.

### 3.6 Exclusion/thresholding analyses

**Fig. 4.**
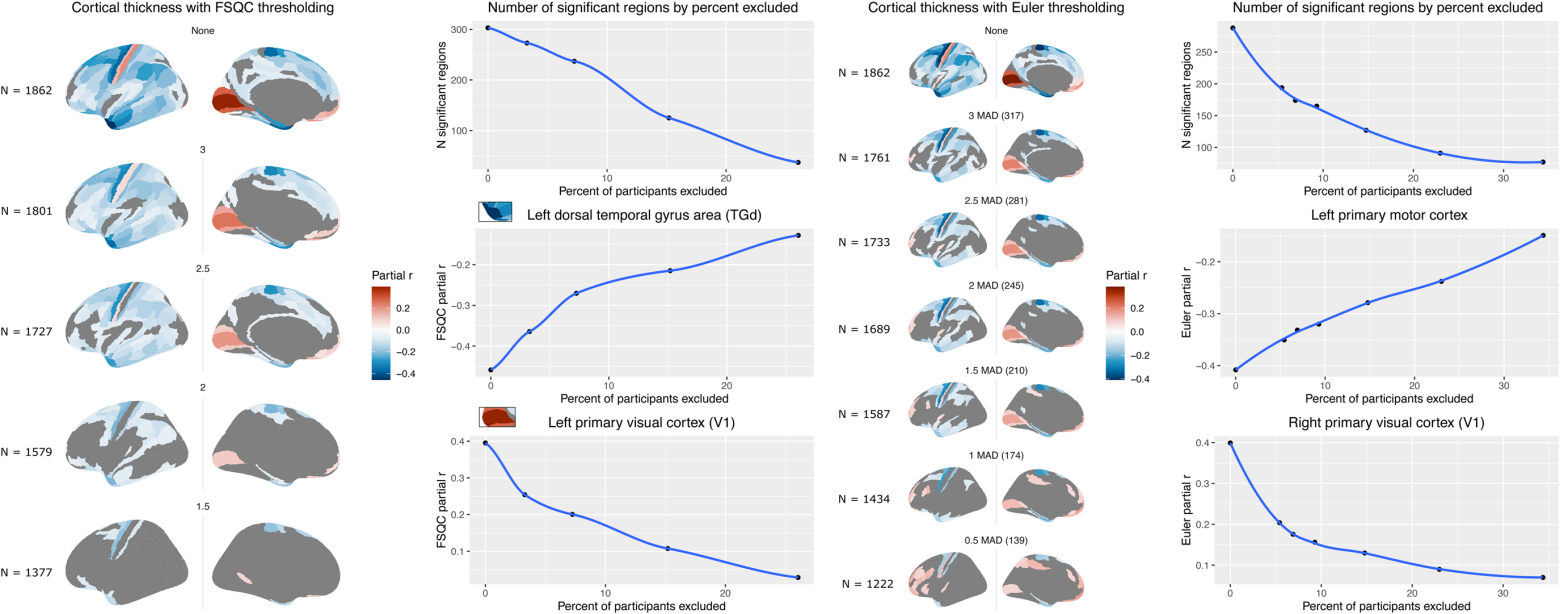
Relationship between cortical thickness and FSQC (left) and Euler number (right) after thresholding at different levels of stringency. Accompanying graphs show the attenuation of both number of significant regions observed (top) and partial correlation effect size (bottom two panels) as stringency increases.

We next examined the impact of different levels of QC thresholding stringency on the relationship between quality and cortical morphometry, based on FSQC and Euler number. For CT, after excluding only scans with the worst FSQC scores (3 and above), effect sizes for the association with FSQC were attenuated, but significant associations were still observed across much of the cortex, following the same spatial patterns as the non-thresholded analysis. Effect sizes were further attenuated, but with similar patterning (strongest results retained) after excluding those with scores higher than 2.5. After excluding at scores of 2 and 1.5, few regions maintained significant associations with FSQC (inferior frontal and temporal regions, and superior frontal cortex and precentral gyrus; Fig. 4).

In the Euler MAD-based thresholding analysis, we observed similar but slightly less stark differences between cut-off points than with FSQC. For CT, an attenuation of effect size was still observed, but was somewhat more gradual and to a lesser extent than when using FSQC. The maps for cut-off points of between 2-3 MAD looked similar, with more of a substantial drop off in significant regions after a cut-off of 1 MAD (Fig. 4). Additional sensitivity analyses all yielded similar results (Supplementary Figures 3.1-3.3).

SA and CV showed a more stark and immediate drop off in significant effects in the FSQC thresholding analyses (Supplementary Figure S3.4). Interestingly, in the Euler thresholding analyses for SA and CV, rather than an attenuation of significant effects, we observed a change in direction, such that associations with Euler number went from mostly negative to mostly positive after thresholding (Supplementary Figure 3.5).

### 3.7 Interaction between image quality and diagnosis

There were minimal differences in cortical morphometry between autistic and neurotypical controls when not accounting for image quality. Autistic individuals had greater CT in the medial primary visual cortex (V1), and a small region in the medial parietal lobe relative to controls, and thinner cortex in a few small regions in the left superior frontal and inferior prefrontal cortex. The effects of controlling for FSQC and Euler number were similar. In these analyses, right V1 was no longer significant; nor were any of the regions which had shown thinner cortex in autism, with the exception of the inferior prefrontal cortical region. Additionally, after controlling for either QC metric, additional significant effects (greater CT in autism relative to controls) were observed in the superior temporal gyrus. Though not many regions survived FDR in any analysis, when examining subthreshold results, we noted that most of the effects that were diminished or disappeared after controlling for quality were those in which apparent thinner cortex in autistic individuals was observed in the original analysis, suggesting that these results may have been an artefact of poor image quality (in the autistic group in particular).

**Fig. 5.**
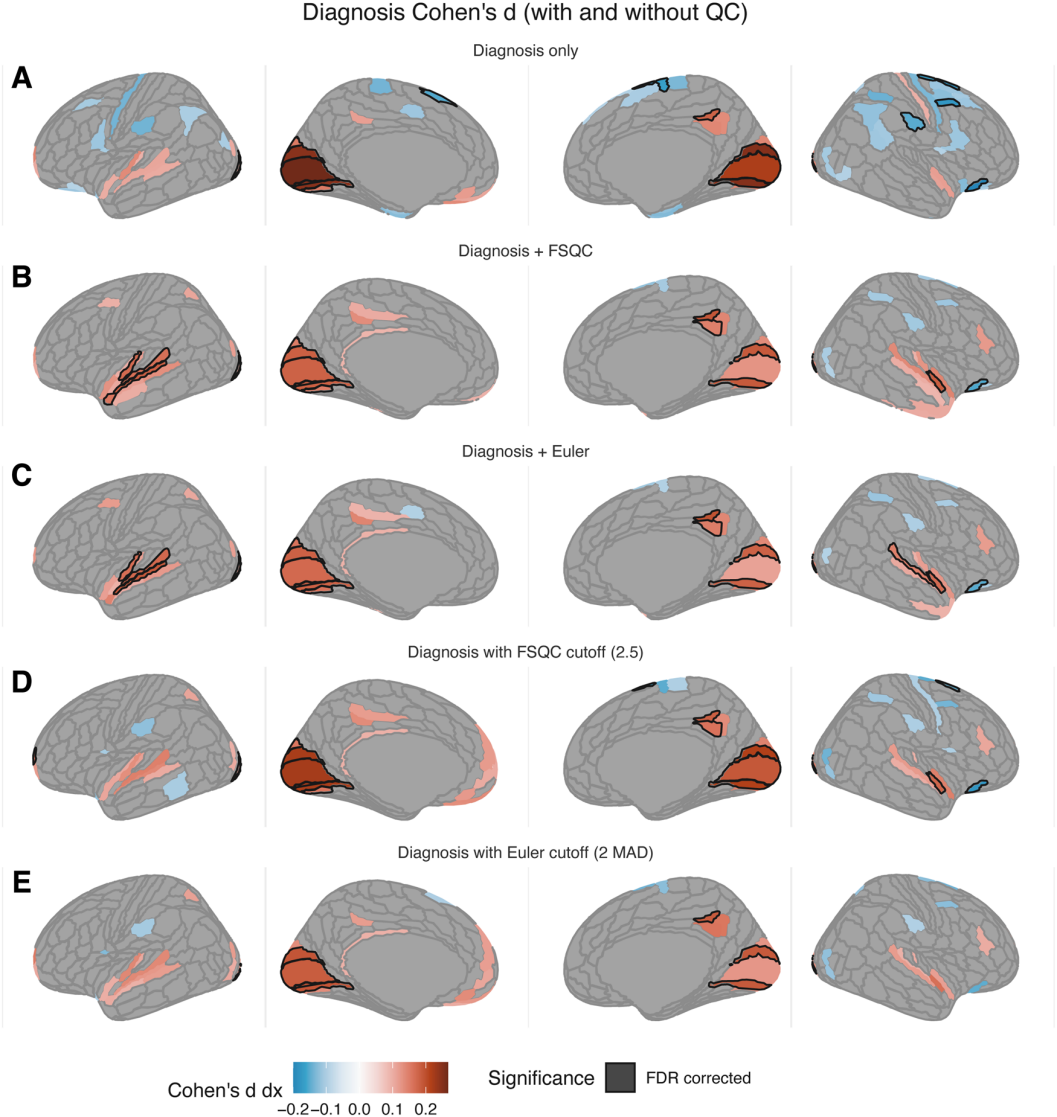
Impact of autism diagnosis on cortical thickness (Cohen’s *d*) without accounting for image quality (A), when controlling for FSQC (B) or Euler (C), and thresholding by FSQC (D) and Euler (E). Significant regions passing 5% FDR are shown with a black border; other regions are subthreshold (i.e., not surviving FDR) differences. Most results indicate thicker cortex in autism relative to controls; results do not change drastically with quality control, but most negative associations between diagnosis and CT (autism < controls) disappear. Significantly thicker cortex in the superior temporal gyrus, which has previously been reported in autism, is observed only when controlling for quality (FSQC or Euler).

Results were very similar, although not identical, after applying QC thresholding (for Euler or FSQC) instead of simply controlling for quality. Results were essentially the same whether applying a cut-off based on FSQC or Euler at a similar stringency (FSQC cut-off of 2.5 [N = 1727]; Euler threshold of 2 MAD or 245 [N = 1689]): only regions in the bilateral medial occipital cortices, and right medial parietal cortex remained significant, all of which were thicker in autism than controls. Again, all regions with thinner cortex in autism were no longer significant after thresholding (Fig. 5). Replication analyses for CT in the POND and CMI datasets yielded largely overlapping results and impact of QC to ABIDE (Supplementary Results S4.1). Results of the main analyses did not change substantially when applying thresholds of different levels of stringency based on FSQC or Euler, though they were slightly further attenuated at each cut-off point (Supplementary Results S4.2).

Combining the two approaches by applying a threshold based on FSQC while also controlling for Euler did not drastically change the results, though some additional regions showed significant associations (Supplementary Figure S4.3). The interaction between quality and diagnosis suggested a stronger relationship between quality and cortical thickness in the autistic group than controls (Supplementary Figure S4.4). Only very minimal group differences in SA and CV were observed, both with and without accounting for image quality (Supplementary Results S4.5).

## 4 Discussion

Our results demonstrate significant, widespread associations between image quality and cortical morphometry across the brain, which are largely consistent across multiple QC metrics. These QC-morphometry interactions persist even after excluding participants with lower image quality, and have marked effects on case-control evaluations. We have outlined several ways to evaluate and correct for the issue of image quality and empirically show that these can improve the robustness of clinical neuroimaging findings.

### 4.1 The FSQC tool enables fast and robust evaluation of image quality in a scalable manner

Our FSQC tool is easy and quick to implement even for large datasets, while still being rigorous and thorough. The generation of multiple images per participant, at multiple orientations and slices across the cortex, allows for a thorough examination of different views without the time-consuming process of individually opening and scrolling through each scan slice by slice. The average rating time per participant of ~20 seconds is also comparable or faster to other published QC protocols (Raamana et al., 2021), and we demonstrate that these times are considerably faster for more experienced raters. We also demonstrate reasonable inter-rater reliability, which is in line with that found by previous studies (Esteban et al., 2017; Klapwijk et al., 2019; Raamana et al., 2021), and demonstrate that different raters do not affect downstream analysis. Importantly, though FSQC primarily assesses quality of FreeSurfer post-processing outputs and surface reconstructions, it also takes into account some aspects of raw image quality (primarily motion). Thus, it can be used either as a complementary tool to existing (perhaps automated) methods, or as a stand-alone tool, simplifying the QC process. Finally, we have shared both our FSQC tool and protocol, and completed image ratings for ABIDE, with the neuroscience community. This could help to save other researchers unnecessary time and effort, and help to improve consistency and reproducibility across studies.

### 4.2 Image quality has largely consistent spatial relationships with cortical morphometry

We demonstrated high correlations and similarity of spatial maps between metrics. This was particularly true for cortical thickness, which also showed the strongest associations. Notably, associations for the automatically generated Euler number and Qoala-T were almost identical to the three manual ratings for cortical thickness, but showed some divergences for cortical surface area and volume. More research is needed to better understand how different QC properties intersect with thresholding or case-control analysis. Here, we focused on FreeSurfer outputs; future studies may want to further explore thresholding based on automated methods such as Qoala-T and MRIQC in multiple datasets (Nakua et al., 2023), as well as how different metrics intersect with sample selection and bias. The striking spatial similarity of FSQC effects with those of motion (both here and in previous work (Alexander-Bloch et al., 2016; Pardoe et al., 2016; Reuter et al., 2015)) confirm that motion is one of the principal sources to impact image quality. With our ratings we provide a comprehensive evaluation of image quality, primarily accounting for the quality of the cortical reconstruction, another important source of bias (Ducharme et al., 2016), but also taking motion into account. We also demonstrate largely consistent effects for cortical thickness across multiple datasets. For cortical thickness, associations with FSQC and Euler were spatially highly similar across four different neurodevelopmental datasets, though they differed in the strength of correlations and number of regions reaching significance (after FDR correction). Some reasons for these differences could include sample composition, demographic differences in cohorts, or differences in scanner type and acquisition parameters. However, we note that the most affected regions, and the directionality of effects, is largely consistent, indicating that the conclusions drawn here are likely to be generalisable across datasets.

Consistent with previous studies (Alexander-Bloch et al., 2016; Ducharme et al., 2016; Gilmore et al., 2021; Pardoe et al., 2016; Reuter et al., 2015; Rosen et al., 2018), we observed largely negative correlations between all three cortical phenotypes and image quality in most brain regions, with a few exceptions. In the case of motion, this is thought to be primarily due to reduced grey-white matter contrast and blurring of the cortical boundary, resulting in incorrect surface reconstruction and, typically, underestimation of cortical thickness (Pardoe et al., 2016; Reuter et al., 2015). Inaccurate surface reconstruction seems to have a similar effect (Ducharme et al., 2016). Cortical volume and surface area estimates seem to be more robust to these types of errors, likely due to the fact that the GM-WM boundary is more impacted than the pial surface, and consequently SA (which relies on the GM-pial surface boundary) and volume (which is a product of SA and CT) show less of an effect of image quality (Pardoe et al., 2016). Indeed, spatial maps for cortical volume were similar to those for thickness, but with weaker relationships, and those for surface area were further attenuated still, with a few key spatial differences. This highlights the importance of careful consideration of which cortical phenotypes are considered in any analysis, in light of evidence that cortical volume and surface area may be more reliable than thickness measures. It also suggests that while careful QC is always important, it may be particularly critical when considering cortical thickness measures versus other cortical phenotypes.

Importantly, these effects were not uniform across the cortex, with some regions being far more susceptible to image quality impacts than others, and some differing in directionality of effects, consistent with previous findings (Alexander-Bloch et al., 2016; Gilmore et al., 2021; Pardoe et al., 2016; Reuter et al., 2015; Rosen et al., 2018). Cortical volume and surface area largely showed similar spatial patterning, though with more positive relationships than CT, particularly for SA, Euler number, and Qoala-T. Some of the regions in which the strongest effects were observed, including the visual cortex, the temporal pole, and primary motor regions, are known to have unique morphometry which may render them more susceptible to issues with image quality and inaccurate surface reconstruction (Scholtens et al., 2015). The temporal and frontal poles are also regions known to have questionable signal quality (McCarthy et al., 2015). Other regional variations in the strength of relationship may in part be attributable to spatial differences in the magnitude of displacement caused by in-scanner head movement, due to participant positioning and restraints or cushioning (Alexander-Bloch et al., 2016). Another factor appears to be the thickness of the region, with higher rates of surface reconstruction errors in areas with thinner cortex causing artificially inflated thickness values (Pardoe et al., 2016). Thus, particular care should be given to interpretation of results for regions which are demonstrably susceptible to image quality.

### 4.3 Thresholding analyses

Consistent with previous work (Ducharme et al., 2016; Gilmore et al., 2021; Reuter et al., 2015), effects of quality were significantly attenuated, but not removed, when excluding participants above a certain cut-off and in a progressive thresholding manner. Excluding participants with the worst image quality may be necessary to limit the impact of bad image quality, though it will likely not remove its impact entirely. The progressive thresholding effects were quite similar for both FSQC and Euler. For Euler, the initial drop off in number of significant regions remaining after QC occurred more quickly but subsequently tapered off, whereas for FSQC the drop off began more gradually, but less significant regions remained after the most stringent threshold than for Euler. In the supplementary Euler percent thresholding analyses, an inflection point for the number of significant regions remaining occurs around 20%, tapering off thereafter. The decrease was more gradual with MAD thresholding. Notably, the speed of attenuation of effect size with increasing QC threshold also varied by region. Thresholding is a balancing act between decreasing the impact of noise and retaining meaningful sample representation and sufficient statistical power and thus may not be appropriate in all contexts. However, our analysis shows that even a minimal threshold can greatly improve the reliability of subsequent downstream results.

### 4.4 Image quality affects case-control differences

Importantly, the effect sizes for quality are, on average, far greater than those of diagnosis, which is concerning in light of evidence that autistic individuals (and those with other clinical diagnoses) tend to move more and have worse image quality than neurotypical controls, in our dataset as well as others (Alexander-Bloch et al., 2016; Pardoe et al., 2016). Thus, there is a high risk of the effects of image quality overshadowing potential diagnostic or group differences, in particular given the finding that the relationship between CT and quality was stronger in the autistic group (likely due to the greater range in quality). In our case-control comparisons, we observed subtle but significant differences depending on the extent and manner in which we controlled for image quality. Many of these differences were consistent across ABIDE as well as the two replication datasets. This is particularly true when observing subthreshold results; regions passing FDR correction differed somewhat, likely owing to differences in sample size, but spatial patterning was largely overlapping. Notably, when not accounting for QC in any way, some significant negative differences were observed (i.e., lower CT in autistic compared to neurotypical individuals), although not all of these survived FDR correction. After accounting for QC, these negative associations were diminished, while the positive associations (i.e., greater CT in autism than controls) were strengthened. This was again consistently observed across all three datasets. Similar effects have been reported previously (Bedford et al., 2020). This is unsurprising given that apparent cortical thinning is known to occur with decreased quality across much of the cortex, coupled with poorer image quality and more motion in autistic individuals. This further underscores the importance of appropriate quality control procedures for case-control analyses.

The results of the diagnosis analyses were largely consistent when controlling for FSQC or Euler at thresholds equating to approximately the same level of stringency, with only very minor differences. Results were also largely consistent when thresholding by QC score cut-off and when controlling for QC score in the analysis. However, a discrepancy was the emergence of significant differences (greater thickness in the autistic group than controls) in the left superior temporal gyrus when including either measure as a covariate, but not when thresholding, in the ABIDE sample. In the absence of a gold-standard ground truth, it is interesting to note that this is a region that has often been implicated in autism in previous work (Bedford et al., 2020; Ecker, 2017; Jou et al., 2010), as well as in the two replication datasets post-QC (with effects in the same direction). It should also be noted that one region that is consistently significant in the case-control comparisons is the occipital cortex (across all three datasets), which is also one of the regions in which we observe the strongest relationship with image quality. Although the effect size is attenuated once QC is accounted for, it remains significant in most of the analyses.

Little work has previously examined the impact of QC on our ability to detect group differences or alterations related to specific diagnoses or conditions. However, several reports of the impact of QC on the effects of age and trajectories of neurodevelopment (Ducharme et al., 2016; Rosen et al., 2018; Savalia et al., 2017) have demonstrated the potential for quality to influence relationships between neuroanatomy and demographic variables of interest. More specifically, motion and other aspects of quality have been demonstrated to both inflate and obscure relationships between age and cortical thickness, and to influence the shape of developmental trajectories (Alexander-Bloch et al., 2016; Ducharme et al., 2016; Rosen et al., 2018; Savalia et al., 2017). The effect sizes for age are typically still larger than those for quality, and therefore unlikely to completely account for previously reported age effects (Alexander-Bloch et al., 2016); however, it may lead to the exaggeration of apparent developmental effects, or ageing-related cortical thinning or atrophy. Moreover, as we have demonstrated, when it comes to diagnostic differences, effect sizes are often subtle and small compared to the relatively strong effects of motion and quality; thus, extra care and attention to QC must be paid when studying neurodevelopmental and psychiatric conditions.

### 4.5 Balancing options for accounting for quality in neuroimaging studies

The trade-off between manual and automated QC (e.g., here, we focused on the comparison of FSQC and Euler number) will of course be up to each individual researcher and dependent on multiple factors relevant to the specific project. We note that there is no widely accepted threshold for Euler denoting good versus poor-quality data; thus, it may be better used in combination with other QC methods. We also note, however, a few key similarities and differences that may be relevant in making this decision. For cortical thickness, the strength and spatial patterning of relationships with FSQC and Euler number were extremely similar. Results of thresholding by various cut-off points using either metric also yielded very similar results for CT, though the number of significant regions dropped off slightly more quickly when using Euler number. The relationships with cortical volume and surface area show more differences between FSQC and Euler number, in particular in the thresholding analyses, with more positive associations observed with Euler number than with FSQC. However, for all cortical phenotypes, thresholding by either measure on the case-control comparison yields very similar results. Thus, multiple factors including the goals of the project, the phenotypes examined, and level of stringency desired will inform the decision between using manual or automated QC methods, or indeed a combination of the two.

Beyond deciding which tools to use, we have discussed and presented two main ways of accounting for quality in analyses: identifying a cut-off point and excluding all participants above or below a specific quality threshold, or controlling for quality scores by including them as a covariate in the statistical analysis. There are benefits and potential pitfalls for both options, and depending on the context one might be preferable to the other. Excluding participants with poor image quality is a common method for QC; however, while this can ensure that the effects of quality are minimised, there are downsides to removing data. First, this necessarily results in a reduction of sample size, and consequently power, which is undesirable particularly considering the cost and effort required to collect neuroimaging data, especially in vulnerable populations. Second, and perhaps more importantly, excluding participants who are likely to have the lowest quality scans introduces unavoidable bias to the dataset: these individuals are likely to be younger and male, and to have a clinical diagnosis, more severe clinical symptoms, and lower IQ (Alexander-Bloch et al., 2016; Bedford et al., 2020; Pardoe et al., 2016). In the context of clinical studies, this can result in samples skewed towards older participants with milder presentations and no intellectual disabilities, thereby potentially excluding participants who could benefit most from research that does not rely on verbal assessment or a minimum IQ (Nordahl et al., 2016). This bias needs to be balanced with the knowledge that poor-quality data may have limited utility or lead to spurious results. Also of note is that image quality in our sample varied significantly by site, highlighting the importance of properly accounting for site effects in multi-site analyses. As has been noted by previous work (Esteban et al., 2017), scanner hardware and sequences may contribute to quality; thus, there is unlikely to be a universal quality threshold that is applicable to all datasets, and this will need to be determined for each individual study.

An alternative solution is to retain all participants, and instead to control for QC by including quality scores as a covariate in the analysis. This avoids some of the above-mentioned biases, but introduces alternate problems. First, retaining all scans regardless of quality risks skewing results, and simply including quality as a covariate is unlikely to account for extreme values in the case of very poor-quality scans. Another issue is the potential for collider bias, occurring when an independent and dependent variable both influence a third variable which is controlled for in an analysis, leading to an apparent (but spurious, or inflated) association (Holmberg & Andersen, 2022; Munafò et al., 2018). In this case, controlling for quality could influence the association between diagnosis and cortical morphometry. However, selection bias can also be considered a form of collider bias, thus this is an issue that should be taken into account regardless of the QC mitigation method chosen.

Finally, to balance pros and cons and harmonise approaches, a hybrid solution can be implemented, whereby only the worst scans which are considered unusable are excluded, and QC is included in the model to correct for any residual effects caused by other lower quality, but still potentially usable, scans. This method could also include a combination of QC tools and metrics; for example, using an automated tool to identify and exclude the lowest quality images, and a manual tool to rate the remaining images.

### 4.6 Limitations

These results should be interpreted in light of certain limitations. First, no quality metric is perfect, and as mentioned above there is no gold standard. Without prospective motion trackers installed at the time of scanning, we cannot accurately quantify motion, and all visual inspections of scan and surface reconstruction quality will have some level of subjectivity. We attempt to mitigate this by comparing multiple QC metrics, both automated and manually rated, by multiple independent raters. Next, we rely on two metrics, FSQC and Euler number, which are specific to FreeSurfer, and thus may have limited generalisability. However, our FSQC tool could easily be applied to other processing and surface reconstruction tools. We also focused exclusively on cortical morphometry. Given recent evidence that subcortical structures are also influenced by quality (though potentially to a lesser degree) (Gilmore et al., 2021), extending the current work to subcortical structures, particularly in the context of clinical group differences, could be valuable. Finally, our sample, the ABIDE dataset, comes from multiple sites internationally, combined retrospectively. Though we accounted for this by using both linear mixed-effects models as well as a meta-analytic technique in all analyses, differences between sites could still impact results. ABIDE also consists of a relatively limited demographic, including mostly children and young adults, a substantial proportion of whom have a diagnosis of autism. However, this dataset allowed us to examine the impact of quality on case-control differences, and we successfully replicated at least some of our results in a much larger, more representative sample.

## 5 Conclusion

Our results highlight the importance of careful quality control of neuroimaging data, and some of the potential consequences of failing to do so. We explored the effect of various QC metrics and mitigation techniques, and demonstrated that these can have a significant impact on our ability to detect differences in neuroanatomy related to autism.

## Supplementary Material

Supplementary Material

## Data Availability

The imaging rating tool, code to generate QC png images and analysis scripts are available at: https://github.com/sbedford0/FSQC. Our ABIDE FSQC ratings are also available at: https://github.com/sbedford0/FSQC/tree/main/ABIDE_ratings. The full protocol can be found at: https://dx.doi.org/10.17504/protocols.io.kxygx9m6wg8j/v1.
